# Templated interfacial synthesis of metal-organic framework (MOF) nano- and micro-structures with precisely controlled shapes and sizes

**DOI:** 10.1038/s42004-021-00522-1

**Published:** 2021-06-03

**Authors:** Lingyao Meng, Binyu Yu, Yang Qin

**Affiliations:** 1 Department of Chemistry and Chemical Biology, University of New Mexico, Albuquerque, NM USA; 2 Department of Chemical and Biomolecular Engineering, Institute of Materials Science, University of Connecticut, Storrs, CT USA

**Keywords:** Organic-inorganic nanostructures, Metal-organic frameworks

## Abstract

Metal-organic frameworks (MOF) are an emerging class of microporous materials with promising applications. MOF nanocrystals, and their assembled super-structures, can display unique properties and reactivities when compared with their bulk analogues. MOF nanostructures of 0-D, 2-D, and 3-D dimensions can be routinely obtained by controlling reaction conditions and ligand additives, while formation of 1-D MOF nanocrystals (nanowires and nanorods) and super-structures has been relatively rare. We report here a facile templated interfacial synthesis methodology for the preparation of a series of 1-D MOF nano- and micro-structures with precisely controlled shapes and sizes. Specifically, by applying track-etched polycarbonate (PCTE) membranes as the templates and at the oil/water interface, we rapidly and reproducibly synthesize zeolitic imidazolate framework-8 (ZIF-8) and ZIF-67 nano- and micro structures of sizes ranging from 10 nm to 20 μm. We also identify a size confinement effect on MOF crystal growth, which leads to single crystals under the most restricted conditions and inter-grown polycrystals at larger template pore sizes, as well as surface directing effects that influence the crystallographic preferred orientation. Our findings provide a potentially generalizable method for controlling the size, morphology, and crystal orientations of MOF nanomaterials, as well as offering fundamental understanding into MOF crystal growth mechanisms.

## Abstract (web summary)

Control over the morphology of porous materials is key for many applications, but can be challenging when targeting small particle sizes. Here one dimensional nano- and microstructures of zeolitic imidazolate frameworks are prepared using track-etched polycarbonate membranes as templates for interfacial synthesis.

## Introduction

Metal-organic frameworks (MOFs), as an emerging and rapidly growing class of microporous materials, are assembled from metal ions or metal ion clusters and bridging organic ligands^1–3^. MOFs have attracted enormous research interests in recent years because of the collections of unique properties including high internal surface area, high pore volume, and easily tunable structure, porosity, and surface functionality^4^. These characteristics make MOFs promising candidates for a myriad of applications including gas/liquid separation^5–7^, gas storage^8,9^, catalysis^10–12^, sensors^13,14^, medicine^15–17^, and electronics^18–20^, etc.

Recently, much research attention has been paid to nanoscale MOFs as they have the potential to synergistically combine and realize properties specific to both MOFs and nanostructures. For example, because of the high ratios of exposed active sites over volumes and rapid adsorption/desorption kinetics, MOF nanoparticles have shown enhanced performance over their bulk analogues in areas such as electronics, catalysis, and biomedicines^17,21–27^. In addition, complex super-structures can be produced through a controlled assembly of MOF nanoparticles, which often leads to emerging properties from cooperative interactions among discrete building blocks^28,29^. Such controlled assembly requires the synthesis of monodisperse MOF nanoparticles with uniform sizes and shapes. Since MOF nanoparticles are very difficult to separate by size post-synthetically, confining the size and shape of nanoparticles during the synthesis has been considered a reliable strategy for the production of uniform nanoparticles. In order to achieve effective and controllable preparation of MOF nanostructures, a large number of bottom-up and top-down synthetic methods have been developed^30^, such as fast precipitation or accelerated heating^31,32^, nanoreactor confinement using emulsion systems^33,34^, and coordination modulation via chemically controlling the ligand-metal interactions^35,36^. Most of the current efforts have been focused on the preparation of 0-D MOF nanoparticles and MOF membranes that are in turn 2-D super-structures of 0-D nanoparticles. On the other hand, obtaining 1-D nanoscale MOF crystallites with excellent uniformity is still a major challenge in this field due to the difficulties in realizing well-defined anisotropic growth, as typical solution-based 1-D MOF syntheses very often lead to mixtures of crystals with broad distributions in sizes and morphologies^37,38^.

Zeolitic imidazolate frameworks (ZIFs), which are composed of imidazolate linkers and metal ions, are an extensively investigated subset of MOFs and possess attractive properties including crystallinity, micro-porosity, high surface area, and high thermal and chemical stability^39,40^. Among the early examples of ZIFs, ZIF-8, and ZIF-67, which are constructed from zinc ions (ZIF-8) and cobalt ions (ZIF-67) with 2-methylimidazolate (2-MIM) ligands, have been widely researched for nano-MOF synthesis and the resulting 0-D nanoparticles and 2-D membranes have found applications in catalysis and gas/liquid separation^41–45^. In comparison with 0-D and 2-D nanostructures, the preparation of 1-D ZIF nanostructures has been much less reported. For instance, 1-D ZIF nanowires or nanorods can be synthesized through top-down approaches including electrospinning^46–48^ and electric field-induced self-assembly of ZIF crystals^49^, and bottom-up, solution-based synthesis with the aid of surfactants^50^ and by controlling the concentrations of seeding solutions^51^. There have also been reports on fabricating ZIF nanorods by using more accessible 1-D structures as templates, such as zinc oxide^52,53^, cobalt oxide^54^, cobalt carbonate hydroxide^55^, tellurium^56^, carbon nanotubes^57^, and organic polymers^58^. Most recently, our group reported the preparation of 1-D ZIF-8 nanowires and nanorods, with respective diameters of 30 and 100 nm, through interfacial synthesis templated by commercial track-etched polycarbonate (PCTE) membranes^59^. The obtained ZIF-8 nanorods and nanowires possess uniform widths and lengths, determined by the templates used. Interestingly, we found a pore-size dependent growth of MOF crystals, in which polycrystalline ZIF-8 nanorods are formed in 100 nm pores and single-crystalline nanowires are formed within 30 nm pores, while in both cases, the crystal lattices are preferably oriented with the {110} planes perpendicular to the long axis of nanorods/wires. Thus, our synthetic method represents a facile strategy to access a wide range of MOF nano- and super-structures and to impart controllability on the MOF growth characteristics.

In the current study, we generalize this templated interfacial synthesis method by using PCTE membranes with a diverse range of pore sizes (10 nm, 30 nm, 100 nm, 200 nm, 2 µm, 10 µm, and 20 µm) and membrane thickness (3 µm, 6 µm, and 10 µm), in attempts to synthesize nanostructures of ZIF-8 and ZIF-67. X-ray diffraction (XRD), transmission electron microscopy (TEM), and scanning electron microscopy (SEM) characterizations reveal that nano- and micro-structures of both ZIF-8 and ZIF-67 can be obtained with high confidence, which is determined only by the pore sizes of templates used. Our methodology can thus serve as a versatile synthetic platform and open up new possibilities in the design and preparation of tailor-made 1-D MOF nano- and super-structures.

## Results

### Synthesis of 1-D ZIF-8 and ZIF-67 nano- and micro-structures

**Fig. 1:**
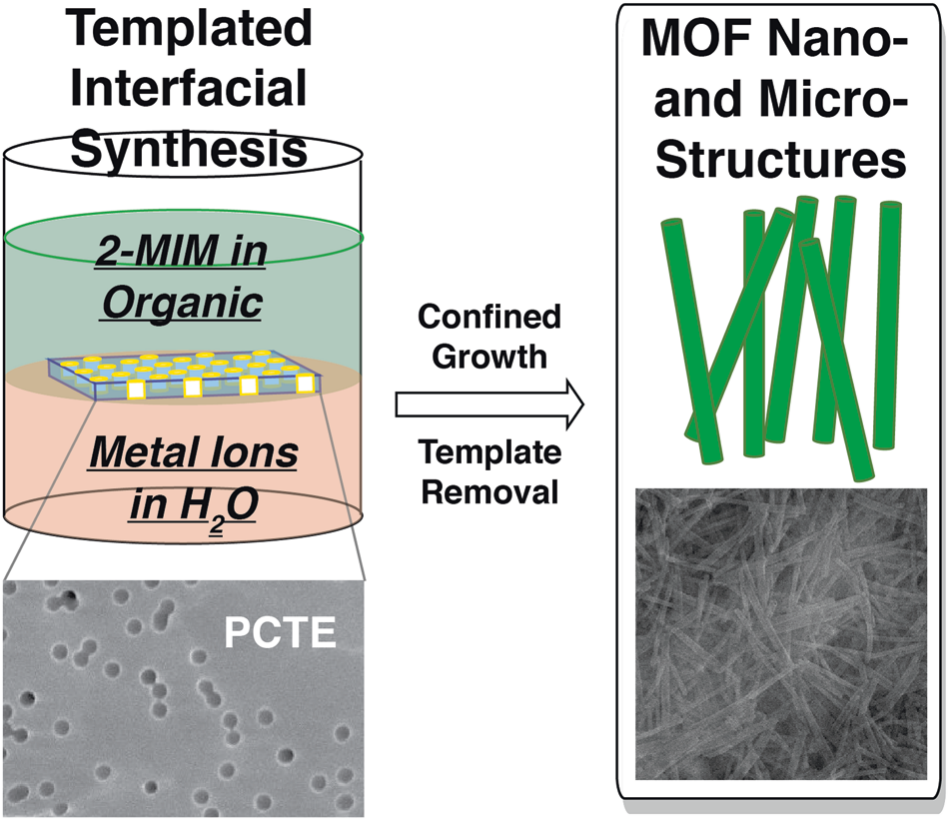
Schematic illustration of the synthesis of ZIF-8 and ZIF-67 1-D nano- and micro-structures. PCET track-etched polycarbonate, 2-MIM 2-methylimidazole.

The track-etched polycarbonate (PCTE) templates are purchased from Sterlitech Corp. Figure S1 of the Supplementary Information (SI) compiles the scanning electron microscopy (SEM) images of both surfaces of the PCTE templates and Table S1 summarizes the manufactory specifications including pore size, template thickness, pore density, and open area percentages. Except for the 10 nm templates, for which we could not observe apparent pore structures likely caused by metal coverage required for SEM measurements, all other templates possess circular pores with uniform diameters matching specifications. It is noted that the pores are randomly distributed and occasionally two or more pores are overlapped to a certain extent, making the pore sizes effectively bigger. It is also noticed that the pores do not align perfectly with some of them having obvious angles deviating from 90° relative to the surfaces. All PCTE templates come with two visually distinct surfaces, one appearing smooth and shinning while the other dull. This visual difference allows us to place these templates the same way during interfacial synthesis as discussed below, having the smooth and shinning surface in contact with water and the dull surface in contact with organic solution. Figure 1 schematically depicts the templated interfacial synthesis toward the ZIF nano- and micro-structures. During a typical synthesis, 10 mL aqueous solutions of Zn(NO_3_)_2_ or Co(NO_3_)_2_ of pre-determined concentrations are placed in a 20 mL scintillation vial, and a PCTE template is gently placed and floats on top of the aqueous solution. A 10 mL solution of 2-methylimidazole (2-MIM) in 1-octanol is then laid on top of the aqueous solution and PCTE template, to initiate the interfacial synthesis. After a certain amount of time, the template is taken out, washed extensively with water, and dried in the air. X-ray diffraction (XRD) and SEM measurements are performed directly on the templates after synthesis to confirm the formation of ZIFs within the template pores. The experimental conditions including concentrations of reactants, which have been shown to significantly impact MOF nano-structure formation processes^60–63^, are systematically adjusted and optimized. The optimization details are included in Tables S6 through S16 and respective XRD and TEM characterization data are included in Fig. S5–S23. It is clear that reactant concentrations play decisive roles in the formation of well-defined MOF nano- and micro-structures, as non-optimized conditions typically led to no formation or formation of only discrete nanoparticles in the template pores. Table S2 lists the optimized conditions that have led to the most well-defined XRD profiles best matching simulated patterns from single-crystal data (see below). The ZIF-8 and ZIF-67 nano- and micro-structures are isolated by dissolving PCTE templates in chloroform and subjected to detailed electron and optical microscopy analyses.

### Characterization on post-synthesis PCTE templates

**Fig. 2:**
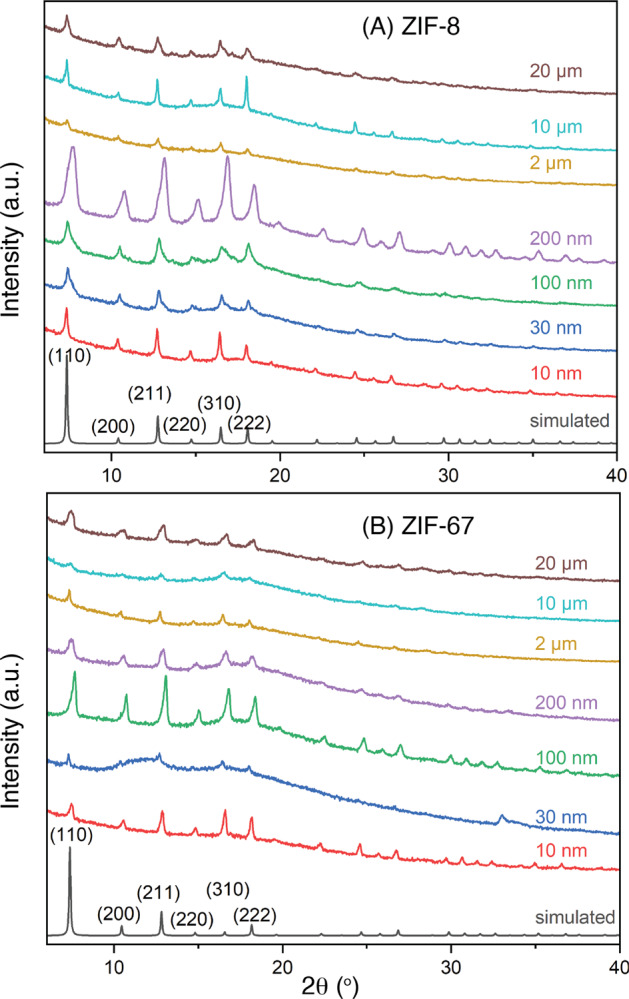
X-ray diffraction (XRD) characterization. XRD patterns of track-etched polycarbonate (PCTE) templates of various pore sizes containing (**A**) ZIF-8 and (**B**) ZIF-67 nano- and micro-structures after interfacial synthesis.

In order to confirm the structural identities of ZIF-8 and ZIF-67 formed within the pores of PCTE templates, we first perform X-ray diffraction (XRD) measurements on the templates immediately after synthesis and the results are compiled in Fig. 2. It is apparent that the XRD profiles of all post-synthesis templates match those simulated from ZIF-8 and ZIF-67 single-crystal data, respectively, thus confirming the formation of corresponding ZIF-8 and ZIF-67 materials within the templates. Signals from the post-synthesis templates appear significantly broader than those simulated and correspond to crystal sizes smaller than those of respective template pores. This is caused by the fact that the MOF materials formed inside template pores are mostly polycrystalline, as discussed below, and are composed of aggregated single crystals that are much smaller than the template pores and thus result in broad PXRD signals. Notably, the relative intensities of the (110) and (200) diffraction peaks from the post-synthesis templates are different than those from corresponding simulated patterns. Such differences have been identified to indicate preferred orientation and growth of MOF crystallites, e.g., ZIF-8 and ZIF-67 have been shown to preferentially grow along and orient the (100) planes parallel to supporting surfaces^64–67^. Such preferred orientation is typically quantified by crystallographic preferred orientation (CPO) indices that are ratios of intensity ratios of a pair of diffraction signals from the subject of interest over those from single crystals. We have thus calculated the CPO indices of (200)/(110) and (200)/(211) Bragg planes, and the results are summarized in Table S3. For ZIF-8, the CPO_(200)/(110)_ indices range between ca. 5 and 8, and the CPO_(200)/(211)_ indices between ca. 1 and 2. As for ZIF-67, the CPO_(200)/(110)_ indices are slightly larger at ca. 7 to 12 and the CPO_(200)/(211)_ indices are between ca. 1 and 3. These observations suggest the preferred orientation of the {100} planes in both ZIFs parallel to the surface of the pore walls, which is in agreement with previous reports although the CPO indices in our cases are significantly smaller. The relatively smaller values of CPO indices are likely caused by the misalignment of the pores created by track-etching, confined growth within cylindrical pores that have large curvatures and may exert less orientation directing effects than do flat surfaces, and residual randomly oriented ZIF nanocrystals on template surfaces as observed in SEM analyses (see below).

**Fig. 3:**
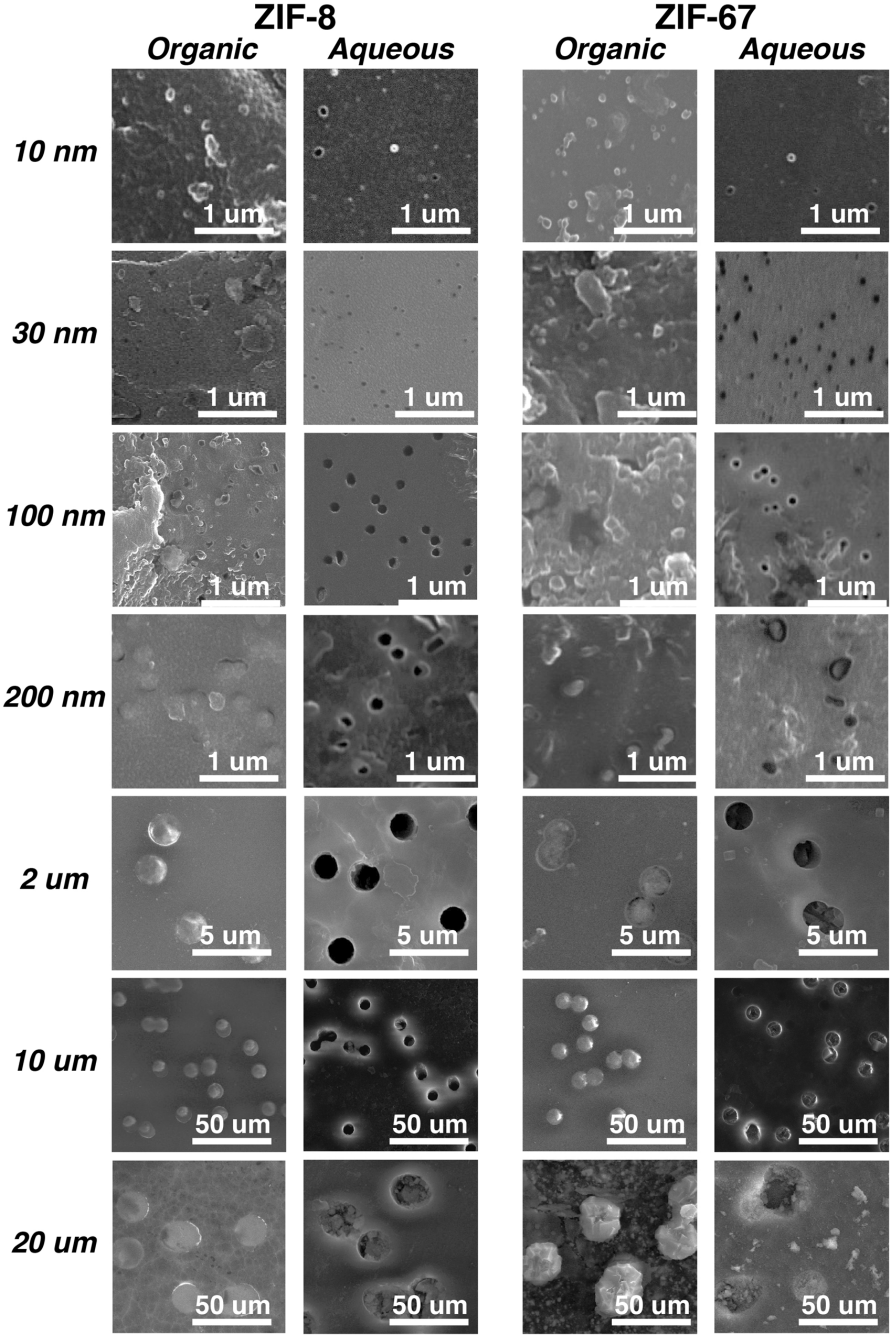
Scanning electron microscopy (SEM) analyses. SEM images of both organic and aqueous facing surfaces of track-etched polycarbonate (PCTE) templates of various pore sizes containing ZIF-8 and ZIF-67 nano- and micro-structures after interfacial synthesis.

We next examine the surface morphologies of post-synthesis templates by SEM and images of both surfaces respectively in contact with organic and aqueous sides are shown in Fig. 3. A similar trend can be observed for both ZIF-8 and ZIF-67. Looking from the sides in contact with organic solutions, the template pores appear to be filled up with solid ZIF crystals in all cases, and these crystals seem to extrude above the surfaces to somewhat extent while maintaining their circular shapes. Randomly distributed ZIF nano-crystals are found on several organic facing surfaces, most prominently on 100 nm ZIF-8, 30 nm ZIF-67, and 100 nm ZIF-67, which may contribute to decreasing the CPO indices discussed earlier. On the other hand, the aqueous facing surfaces look generally cleaner with less surface-bound nanocrystals, and the pores seem empty, partially filled, or completely filled depending on the ZIF types and pore sizes. For ZIF-8, the 30 nm, 100 nm, and 2 μm pores seem to be empty, while solid objects can be seen in some of the 200 nm and 10 μm pores. For the 20 μm pores, it is obvious that they are filled up with ZIF-8 crystallites and contrary to the organic facing sides, the crystallites are recessed from the surfaces to certain degrees. For ZIF-67, the pores seem to be filled up more regularly. No crystallites are obvious in the 30 nm pores, while the 100 nm pores seem to be partially filled. Pores of other sizes appear to be filled up with ZIF-67 crystallites that are recessed to various degrees from the surfaces. As for the 10 nm post-synthesis templates, uniform dots, and empty circles can be observed respectively on the organic and aqueous facing surfaces for both ZIFs, but their sizes seem to be significantly larger than 10 nm. We are not certain about the causes for such observations at the current stage. In short summary, in combination with XRD and SEM characterization, our templated interfacial synthesis produces ZIF-8 and ZIF-67 crystallites within the template pores, and some of these crystals reach both surfaces of the templates, suggesting that the structures traverse the entire thickness of templates. Thus, we next attempt to isolate these nano- and micro-structures by the dissolution of PCTE templates so that we can directly visualize their shapes and sizes.

### Characterization of isolated ZIF-8 and ZIF-67 nano- and micro-structures

**Fig. 4:**
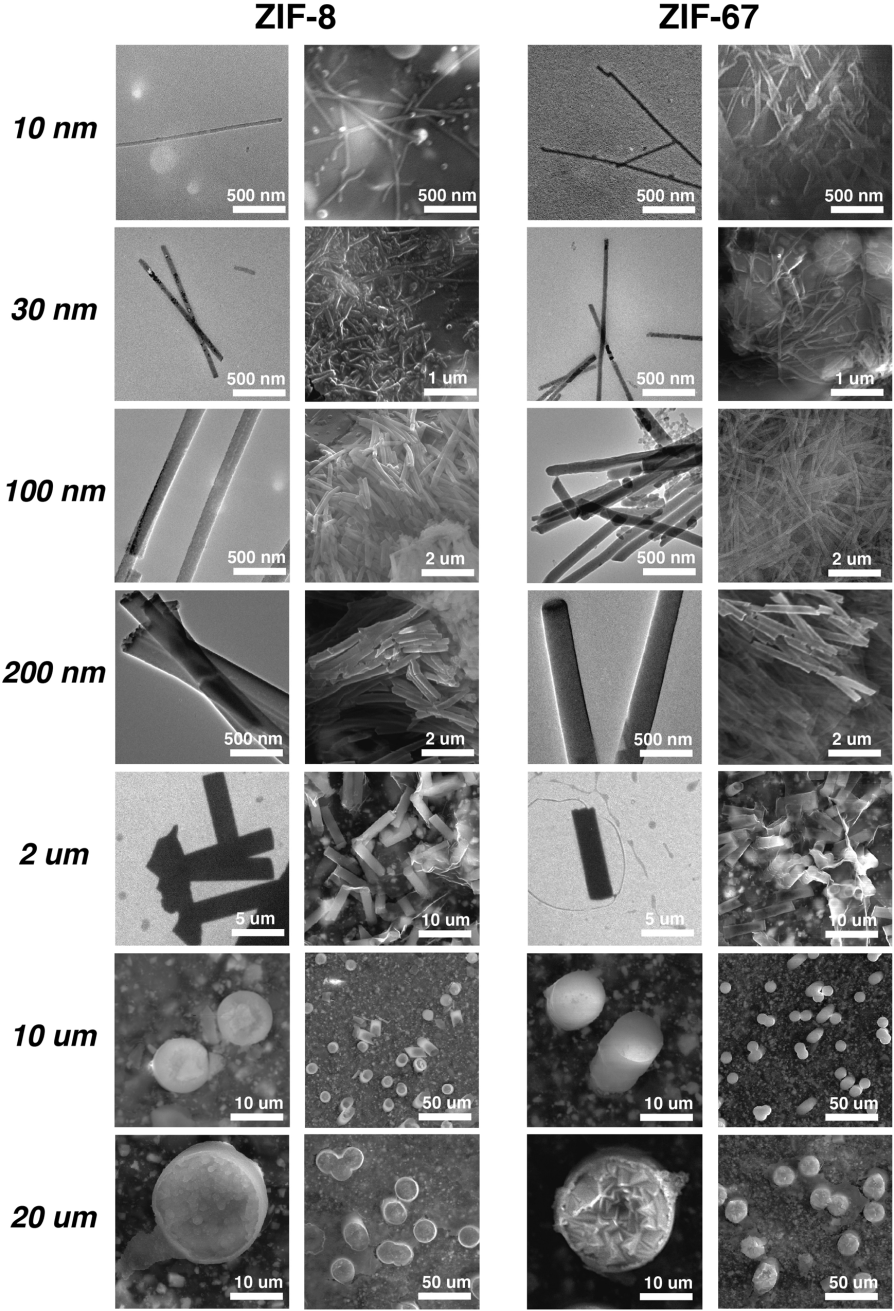
Electron microscopy images of isolated nano- and micro-structures. Isolated structures by dissolution (left columns) and on conducting tape supports (right columns) for both ZIF-8 and ZIF-67, respectively. For dissolved structures, images for sizes 10 nm to 2 μm are from transmission electron microscopy (TEM), while images for sizes 10 and 20 μm are from scanning electron microscopy (SEM). Structures on conducting tape supports are all analyzed by SEM.

**Fig. 5:**
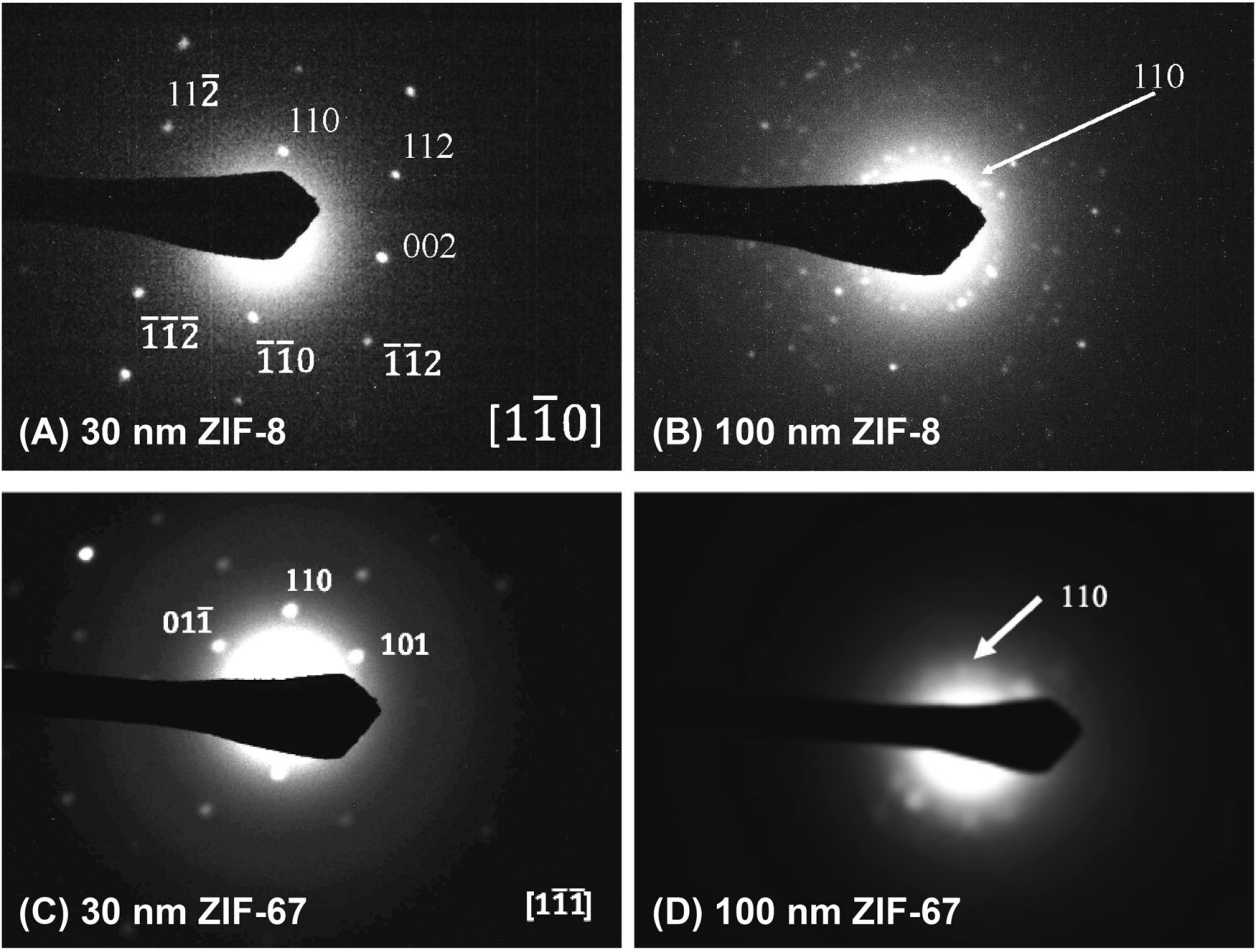
Selected area electron diffraction (SAED) patterns. **A** 30 nm ZIF-8 nanowire, (**B**) 100 nm ZIF-8 nanorods, (**C**) 30 nm ZIF-67 nanowires, and (**D**) 100 nm ZIF-67 nanorods.

We apply two different protocols for isolating the ZIF nano- and micro-structures. The first is to dissolve the PCTE templates in chloroform and drop cast the solutions onto either carbon-coated grids for TEM measurements or onto glass slides for SEM and optical microscopy analyses. We refer to these isolated nano- and micro-structures as dissolved structures in the following discussions. On the other hand, we attach the post-synthesis PCTE templates to glass substrates from the organic sides by using J–B Weld, then the PCTE templates are removed by washing with tetrahydrofuran followed by metal coating and then SEM analysis. For clarification, we call nano- and micro-structures isolated this way the sticked structures. The results are compiled in Figs. 4 and 5, Fig. S2, and Table S4. In Fig. 4, the left columns for ZIF-8 and ZIF-67 are dissolved structures, while the right columns are respectively sticked structures. For both left columns, the images for sizes from 10 nm to 2 μm are from TEM measurements, and those of 10 μm and 20 μm are SEM images. All images for the sticked structures are from SEM analyses. Figure 5 includes the selected area electron diffraction (SAED) images of 30 nm nanowires and 100 nm nanorods of ZIF-8 and ZIF-67, respectively. In Fig. S4, additional images are provided for the dissolved structures, in which TEM is used for sizes from 10 to 200 nm while optical microscopy for 2, 10, and 20 μm samples. Table S4 summarizes the geometric parameters, including diameters and lengths, of isolate nano- and micro-structures for both ZIFs by sampling 100 individual dissolved structures in each size category.

It is clearly seen that continuous and uniform nanowires (10 and 30 nm), nanorods (100 and 200 nm), micro rods (2 μm), microcylinders (10 μm), and microdisks (20 μm) of ZIF-8 and ZIF-67 are created, the diameters of which generally match those of the template pores. The exceptions are 10 nm ZIF-8 and ZIF-67 nanowires, for which we have not been able to visualize empty pore sizes of the PCTE templates and the measured diameters of nanowires are significantly bigger than 10 nm. It is also noted that the average diameter of 100 nm ZIF-8 is also quite larger than that of the template pores, for which we do not currently know the causes. On the other hand, lengths of the ZIF nano- and micro-structures are generally smaller than the thickness of PCTE templates, except for the 10 and 20 μm structures that have lengths comparable to the thickness of the templates and 10 and 3 μm, respectively. This observation is on one hand in agreement with the SEM analyses on post-synthesis templates (Fig. 3), where template pores of smaller sizes do not look filled from the aqueous sides; while on the other hand, ZIFs are known to be brittle materials and these newly prepared ZIF nanowires, nanorods, and micro rods possess relatively large aspect ratios and can be easily broken during the isolation processes, thus effectively reducing apparent lengths. We also attempted SAED measurements on the dissolved ZIF nano- and micro-structures and could only be able to obtain patterns for the 30 and 100 nm samples as shown in Fig. 5. MOFs are known to be sensitive to electron beams that cause structural damages^68^, which may explain why we could not obtain SAED patterns for 10 nm ZIF nanowires. On the other hand, structures of larger dimensions are likely too thick for electron diffraction into meaningful patterns. As shown in Fig. 5, 100 nm nanorods of both ZIF-8 and ZIF-67 are polycrystalline, or super-structures in inter-grown ZIF nanocrystals. It is expected that the nano- and micro-structures of large sizes are also super-structures of ZIF nanocrystals, which is most clearly seen in the SEM images of 20 μm ZIF-8 and ZIF-67 microdisks (Fig. 4). Intriguingly, the SAED patterns suggest that both the 30 nm ZIF nanowires are single-crystalline with well-defined scattering patterns, in which the electron beam directions align with the $[1\bar 10]$ and $[1\bar 1\bar 1]$ axis of ZIF-8 and ZIF-67 nanowires, respectively. This observation suggests different crystal growth mechanisms for the same MOF materials under different size-confinement conditions.

## Discussion

**Fig. 6:**
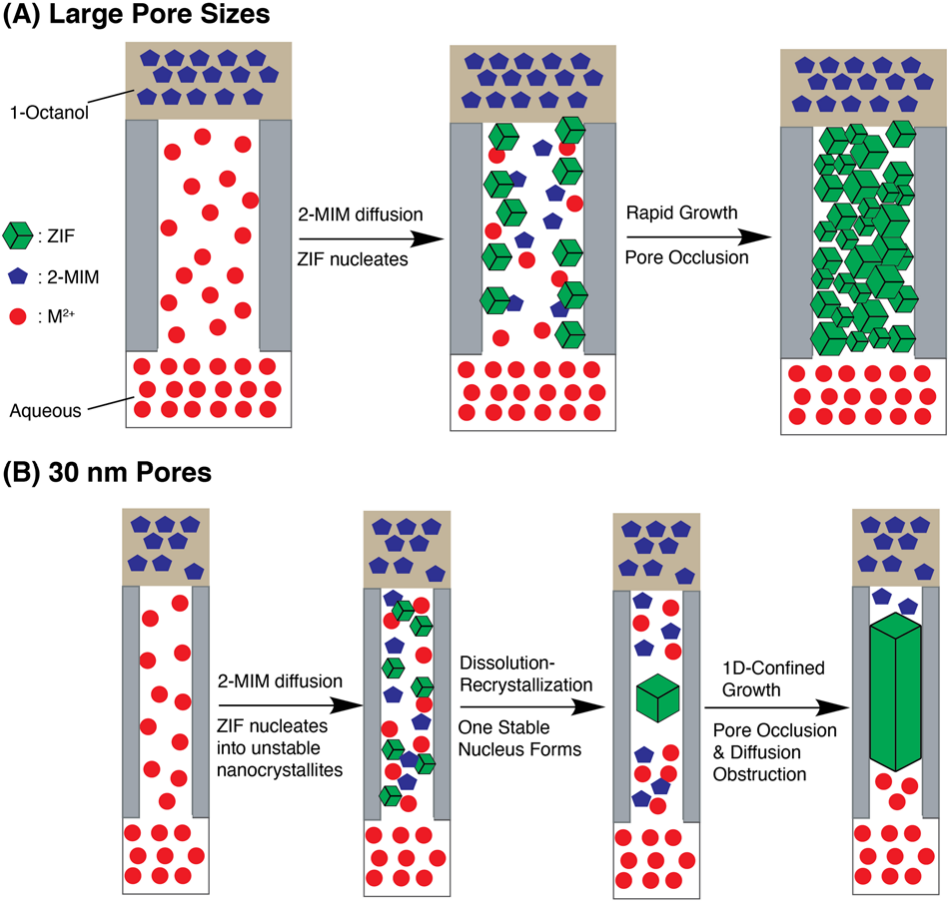
Proposed ZIF growth mechanisms. Growth mechanisms inside the template pores of (**A**) larger than 30 nm, and (**B**) 30 nm in diameter.

The previous synthesis of MOF nano- and super-structures using nanoporous templates, such as anodic aluminum oxide and polymer membranes, via interfacial or counter-diffusion manners exclusively led to the formation of 2-D MOF membranes on one side of the templates^69–73^. In our case, the application of PCTE templates results in the formation of 1-D MOF nano- and micro-structures within the pores of the templates. PCTE templates have been extensively applied for the synthesis of 1-D nanostructures of conjugated polymers and metal oxides, in which the poly(N-vinylpyrrolidinone) (PVP) pore surface coatings have been considered as the key factor to confine the synthesis within the pores through hydrophilic, coordination, and/or hydrogen-bonding interactions with the reactants and resulting nanocrystals^74–79^. On the other hand, the Zn^2+^ and Co^2+^ salts are insoluble in 1-octanol, while 2-MIM is readily dissolved in water. Such solubility difference has been shown to significantly influence crystal growth rates^80,81^ and, in our case, leads to selective diffusion of 2-MIM to aqueous phase inside the template pores but not the metal ions to the organic phase, and thus the formation of MOF nano-structures only inside the template pores. We thus contemplate the growth mechanisms as shown in Fig. 6, which are different for ZIFs in 30 nm pores and bigger sized pores. For pores bigger than 30 nm and at the start of the reactions, metal ions and 2-MIM molecules mix inside the pores and initiate the formation of ZIF nanocrystals. The PVP coatings behave as anchors for these nanocrystals through coordination interactions between pyrrolidine units and metal ions. Such interactions align these initially formed nanocrystals along the pore walls and induce further growth inward until the nanocrystals are inter-grown and fill up the pore volume, which stops ion/2-MIM diffusion and thus terminates the reactions. Such interactions also seem to exert the strongest adhesion to the {100} crystal planes, leading to the observed growth orientation preferences. Furthermore, it has been shown that during ZIF crystallization processes, the initially fastest-growing facets are the {100} faces, resulting in cubic seed crystals, followed by the dominating growth of 12 {110} faces, eventually leading to truncated rhombic dodecahedron single crystals^82^. Inside the PCTE template pores, the spatial confinement in two dimensions leads to the fastest-growing {110} faces along the only unrestricted direction, i.e., the pore’s long axis, resulting in the observed CPO. When the template pore sizes reduce to 30 nm, we suspect that the initially produced ZIF seed crystals are no longer stable due to high surface tension at such small sizes. As a result, these nanocrystals undergo a rapid re-dissolution/recrystallization process similar to the Oswald ripening mechanism, eventually leading to one single crystal of the largest size that is along the pore axis.

The interfacial templated synthesis of MOF nano- and micro-structures can be generalized and extended to other types of MOF systems, where reaction conditions need to be adjusted and optimized. For example, we have attempted the synthesis of ZIF-7 nano-rods using the PCTE template with 100 nm pores, from Zn(NO_3_)_2_ and benzimidazole. The reaction conditions including reactant concentrations were optimized and listed in Table S5. Condition (b), i.e., with Zn(NO_3_)_2_ and benzimidazole concentrations at 0.236 and 2 M, respectively, led to the formation of ZIF-7 nano-rods as confirmed by XRD profiles (Fig. S3) and TEM images (Fig. S4). We are currently working on expanding the scope further to a diverse set of MOF systems and will report any findings in future accounts. On the other hand, the interfacial synthesis seems to work well only using track-etched PCTE templates under the conditions discussed. Previous reports on the templated interfacial synthesis of ZIF-8 using porous polymer and anodized aluminum oxide (AAO) membranes exclusively led to the formation of ZIF-8 membranes on one side of the templates without continuous nano-structures inside the pores^34,71,72,83^. We have attempted ZIF-8 synthesis using track-etched PET templates of 200 nm pores from the same commercial source under identical conditions. The cross-section SEM images of both post-synthesis templates are shown in Fig. S24. ZIF-8 nanorods are found inside the PCTE template pores but not in any of the PET pores. Instead, a thick layer of ZIF-8 membrane is found on the side of the PET template facing 1-octanol. Since the commercial track-etched PET templates are also hydrophilic but are not PVP treated, the lack of ZIF nano-structures inside pores indirectly confirms the important functions of PVP as discussed earlier. Thus, we are currently working on treating various porous templates, including PET templates, with PVP in order to confirm its nano-structure anchoring effects.

In summary, we have introduced a facile templated interfacial synthesis methodology for the access of MOF 1-D nano- and super-structures with a wide range of sizes. Our findings reveal a fundamental understanding of the MOF crystal growth mechanisms under surface functionalization and size confinement conditions, which can be invaluable in directing future MOF materials design and synthesis. We expect our technique can be applied to a wide range of MOF materials and potentially composite membranes with tailor-designed and precisely arranged MOF nanostructures.

## Methods

### Materials

Zinc nitrate hexahydrate (Zn(NO_3_)_2_•6H_2_O, Alfa Aesar, 99%), cobalt nitrate hexahydrate (Co(NO_3_)_2_•6H_2_O, Alfa Aesar, 98–102%), 1-octanol (Alfa Aesar, 99%), reagent grade water (BDH), and 2-methylimidazole (2-MIM, Acros Organics, 99%) were used as received without further purification. Track-etched polycarbonate membranes were purchased from Sterlitech Corporation (Kent, WA) and were used as received.

### Synthesis of ZIF-8 and ZIF-67 nanostructures

In a typical synthesis of ZIF-8 nanostructures, predetermined amounts of Zn(NO_3_)_2_•6H_2_O and 2-MIM were dissolved in reagent grade water and 1-octanol, respectively. PCTE membranes were then floated on the surface of the metal-containing aqueous solution with the dull side up for 24 h. Next, the 2-MIM solution was gently layered on the top of the membrane. After predetermined reaction times, PCTE membranes were taken out, rinsed thoroughly with DI water, and dried in air. In the case of synthesizing ZIF-67 nanostructures, Co(NO_3_)_2_•6H_2_O was used instead of Zn(NO_3_)_2_•6H_2_O. The detailed reaction conditions are listed in Table S1.

### Characterizations

Transmission electron microscopy (TEM) samples were prepared by dissolving as-synthesized PCTE membranes in 8 mL chloroform, and then drop-casting onto carbon-coated copper grids (TED Pella Inc.). TEM images and selected area electron diffraction (SAED) patterns were taken on a JEOL 2010F TEM at an acceleration voltage of 200 kV. X-ray diffraction (XRD) patterns were recorded at room temperature using a Rigaku Smartlab diffractometer with a Cu Kα beam (*λ* = 1.54 Å) operated at 40 kV and 40 mA. SEM samples were prepared by cutting PCTE membranes in half and then mounted onto a glass substrate using double-sided carbon tape. A layer of aluminum (about 10 nm thick) was then deposited on top of the membrane using the angstrom Engineering Amond deposition system. Isolated SEM samples were prepared by coating the organic side of the membrane with 10 nm aluminum, and then gluing the hydrophobic side onto a glass substrate using J–B Weld. The resulting samples were then soaked in THF for 10 min, and then taken out, dried in air. Finally, 10 nm of aluminum was deposited on the substrate. Scanning electron microscopy (SEM) images were taken on a FET Quanta 3-D FEG SEM/FIB instrument.

## Supplementary information

Supplementary Information

## Data Availability

The data that support the findings of this study are available from the corresponding author upon reasonable request.
