# Supplementary material for: Templated interfacial synthesis of metal-organic framework (MOF) nano- and micro-structures with precisely controlled shapes and sizes
Source: Commun Chem. 2021 Jun 3;4:82. doi: 10.1038/s42004-021-00522-1 (PMC9814928; doi:10.1038/s42004-021-00522-1)
Supplement: Supplementary file 1 — Supplementary Information [file 42004_2021_522_MOESM1_ESM.pdf]

Supplementary Information

Templated Interfacial Synthesis of Metal-Organic Framework (MOF) Nano- and Micro-Structures with Precisely Controlled Shapes and Sizes

*Lingyao Meng,[1] Binyu Yu[2] and Yang Qin[1,2] **

[1] Department of Chemistry and Chemical Biology, University of New Mexico, Albuquerque, NM 87131, USA
[2] Department of Chemical and Biomolecular Engineering, Institute of Materials Science, University of Connecticut, CT 06269, USA

* Corresponding Author; E-mail: yang.qin@uconn.edu

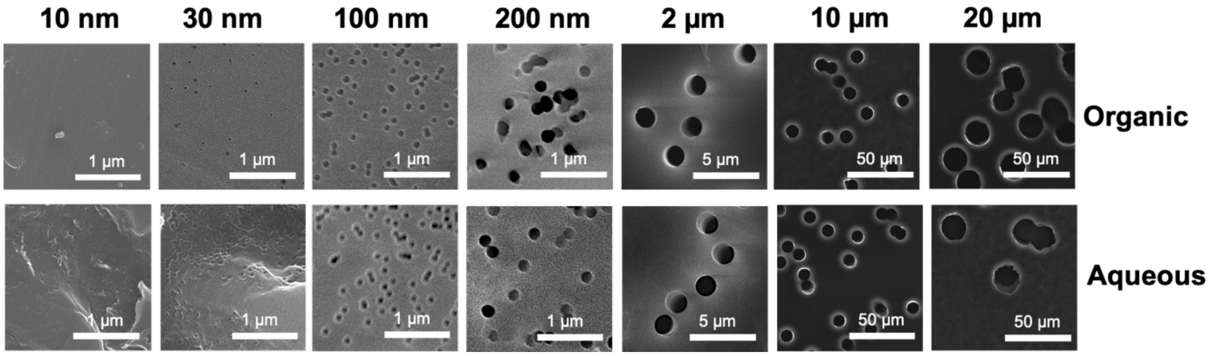

Figure S1: Scanning electron microscopy (SEM) images of both surfaces of pristine track-etched polycarbonate (PCTE) membranes with different pore sizes applied for the templated interfacial synthesis in the current studies. The terms "organic" and "aqueous" indicate surfaces in contact with 1-octanol and water solutions during synthesis, respectively.

Table S1. Manufactory specifications of the track-etched polycarbonate (PCTE) membranes.

Pore Diameter	*Thickness (μm)*	*Pore Density (pores/cm^2)*	*Open Area (%)*
10 nm	6	6 x 10^8	< 1
30 nm	6	6 x 10^8	<1
100 nm	6	4 x 10^8	3
200 nm	10	3 x 10^8	10
2 μm	10	2 x 10^6	6
10 μm	10	1 x 10^5	8
20 μm	3	4 x 10^4	13

Table S2. Reaction conditions for the synthesis of ZIF-8 and ZIF-67 nano- and micro-structures.

ZIF-8/Pore Size	*[Zn^{2+}] Conc.(M)*	*[2-MIM] Conc. (M)*	*Reaction Time (h)*
10 nm	0.06	1	4
30 nm	0.025	1	1
100 nm	0.042	2	1
200 nm	0.025	0.5	4
2 µm	0.1	2	4
10 µm	0.025	1	4
20 µm	0.025	2	4
ZIF-67/Pore Size	*[Co^{2+}] Conc.(M)*	*[2-MIM] Conc. (M)*	*Reaction Time (h)*
10 nm	0.042	2	4
30 nm	0.025	0.75	1
100 nm	0.06	2	1
200 nm	0.06	2	4
2 µm	0.1	2	4
10 µm	0.06	1	6
20 µm	0.1	2	6

Table S3. Crystallographic preferred orientation (CPO) indices for different sizes of MOF nanostructures.

ZIF-8/Pore Size	*CPO (002)/(011)*	*CPO (002)/(112)*
10 nm	6.17	1.26
30 nm	5.25	1.51
100 nm	7.09	1.96
200 nm	5.64	1.38
2 µm	6.14	1.27
10 µm	7.34	1.45
20 µm	7.55	1.98
ZIF-67/Pore Size	*CPO (002)/(011)*	*CPO (002)/(112)*
10 nm	11.28	2.05
30 nm	9.20	2.78
100 nm	10.02	2.13
200 nm	9.49	2.21
2 µm	7.78	2.46
10 µm	10.60	2.08
20 µm	7.89	1.79

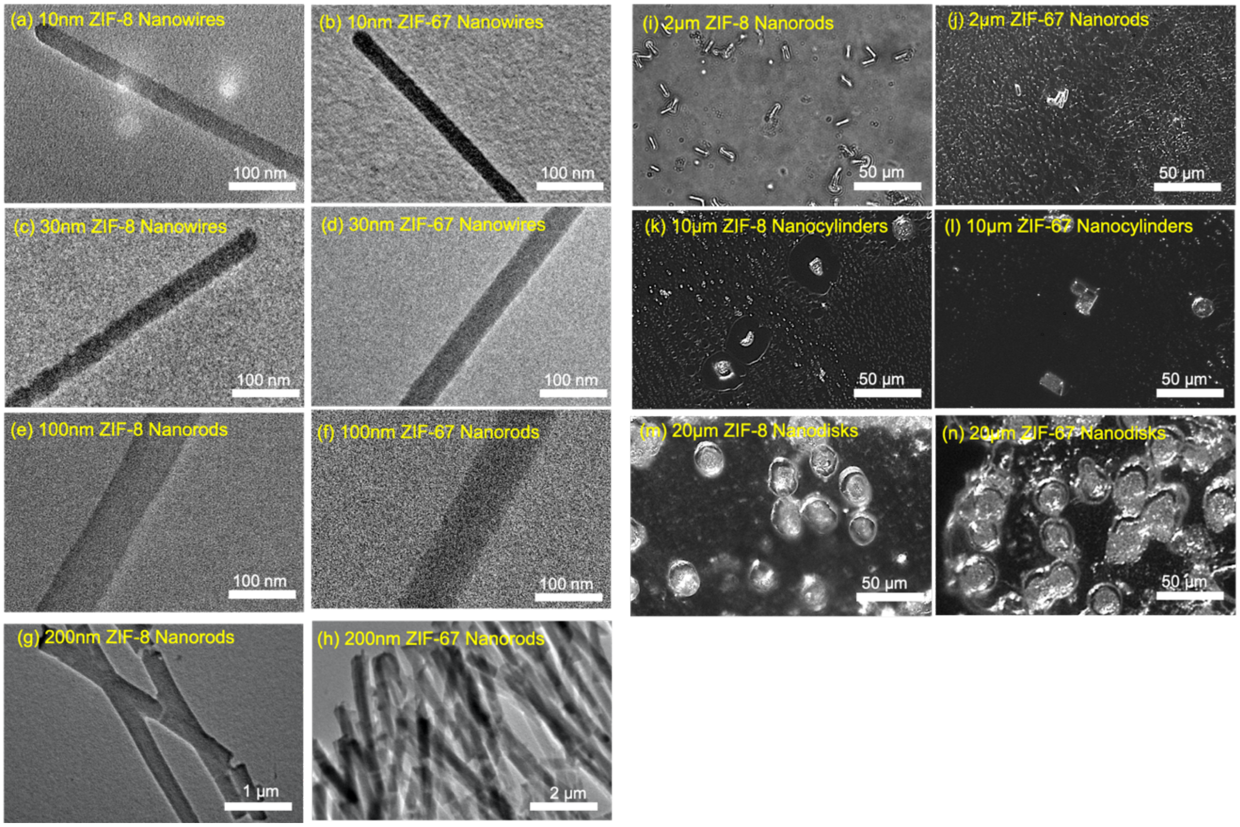

Figure S2. (a)-(h) Transmission electron microscopy (TEM) images of ZIF-8 and ZIF-67 nanowires and nanorods formed within 10 nm, 30 nm, 100 nm and 200 nm pores of corresponding PCTE templates; (i)-(n) optical microscopic images of ZIF-8 and ZIF-67 micro-rods, micro-cylinders, and micro-disks formed respectively in 2µm, 10 µm, and 20 µm pores of corresponding PCTE templates.

Table S4. Template pore diameters, ZIF structure diameters and lengths sampled and averaged from 100 individual subjects of dissolved structures.

Structure	*Template pore diameter*	*Diameter*	*Length*
10 nm ZIF-8	N/A	18.33 ± 2.51 nm	1.74 ± 0.26 μm
30 nm ZIF-8	26.48 ± 4.79 nm	30.53 ± 2.24 nm	1.65 ± 0.69 μm
100 nm ZIF-8	84.24 ± 12.96 nm	112.51 ± 14.30 nm	2.39 ± 0.91 μm
200 nm ZIF-8	197.62 ± 16.23 nm	197.25 ± 24.52 nm	2.60 ± 0.66 μm
2 μm ZIF-8	1.67 ± 0.12 μm	1.58 ± 0.16 μm	7.56 ± 0.59 μm
10 μm ZIF-8	8.13 ± 0.84 μm	7.24 ± 1.07 μm	10.67 ± 2.48 μm
20 μm ZIF-8	19.14 ± 1.67 μm	19.22 ± 1.45 μm	2.38 ± 0.60 μm
10 nm ZIF-67	N/A	24.01 ± 2.62 nm	1.78 ± 0.70 μm
30 nm ZIF-67	26.48 ± 4.79 nm	28.86 ± 2.71 nm	1.38 ± 0.39 μm
100 nm ZIF-67	84.24 ± 12.96 nm	76.01 ± 7.63 nm	1.98 ± 0.37 μm
200 nm ZIF-67	197.62 ± 16.23 nm	204.18 ± 17.26 μm	4.02 ± 0.63 μm
2 μm ZIF-67	1.67 ± 0.12 μm	1.76 ± 0.13 μm	6.92 ± 0.48 μm
10 μm ZIF-67	8.13 ± 0.84 μm	8.20 ± 0.79 μm	9.63 ± 2.37 μm
20 μm ZIF-67	19.14 ± 1.67 μm	18.94 ± 1.35 μm	2.88 ± 0.52 μm

Table S5. Synthesis condition optimization for ZIF-7 nano-structures using PCTE templates with 100 nm pore sizes. BIM: benzimidazole. $Zn(NO_3)_2$ is the Zn^{2+} source.

Attempts	*[Zn^{2+}] Conc.(M)*	*[BIM] Conc. (M)*	*Reaction Time (h)*
(a)	0.236	1	4
(b)	0.236	2	4
(c)	0.236	3	4

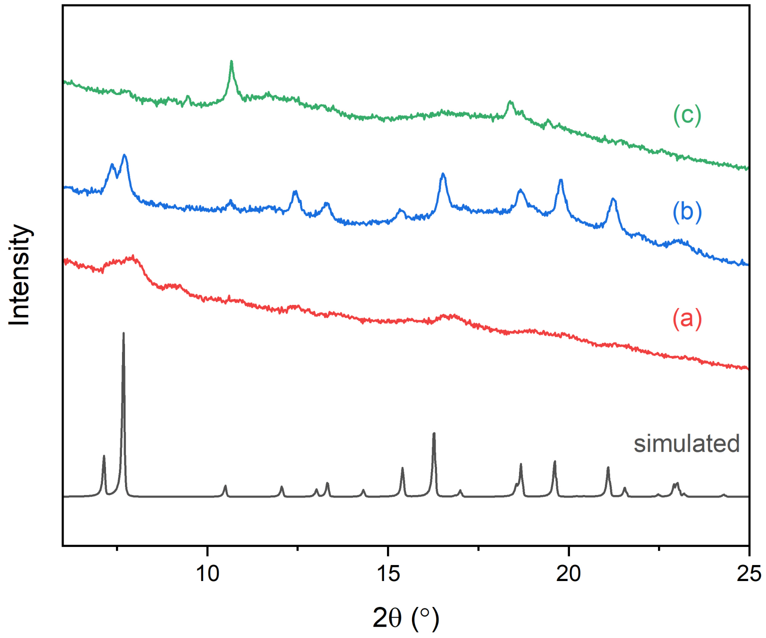

Figure S3. PXRD profiles of post-synthesis templates using conditions listed in Table S5.

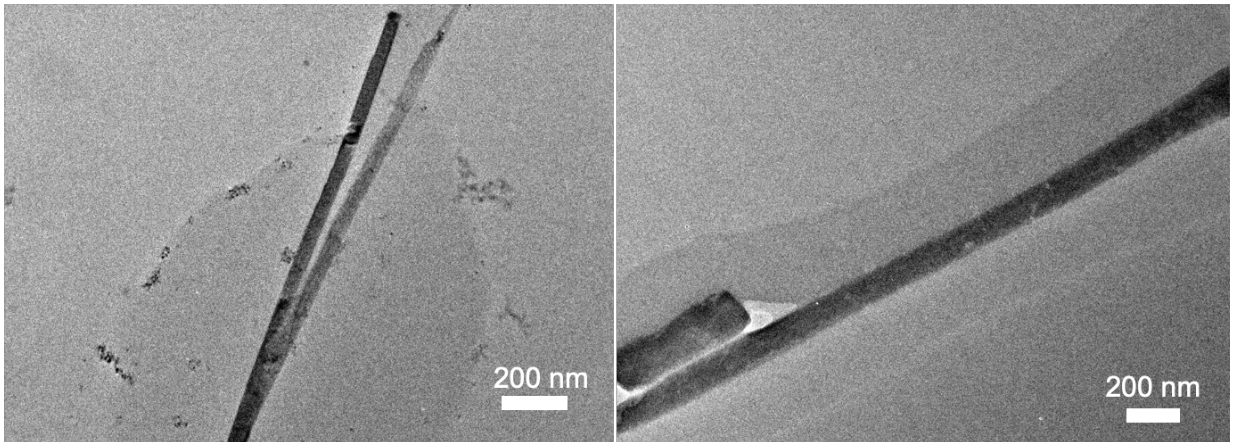

Figure S4. TEM images of isolated ZIF-7 nanostructures from reaction conditions (b) in Table S5.

Table S6. Synthesis condition optimization for ZIF-8 nano-structures using PCTE templates with 10 nm pore sizes. 2-MIM: 2-Methylimidazole. $Zn(NO_3)_2$ is the Zn^{2+} source.

Attempts	*[Zn^{2+}] Conc.(M)*	*[2-MIM] Conc. (M)*	*Reaction Time (h)*
(a)	0.025	1	4
(b)	0.06	1	4
(c)	0.025	2	4

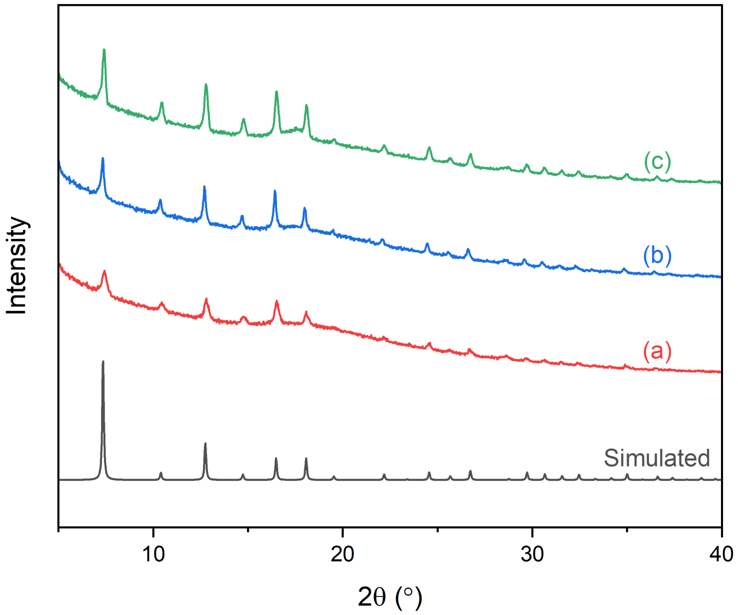

Figure S5. PXRD profiles of post-synthesis templates using conditions listed in Table S6.

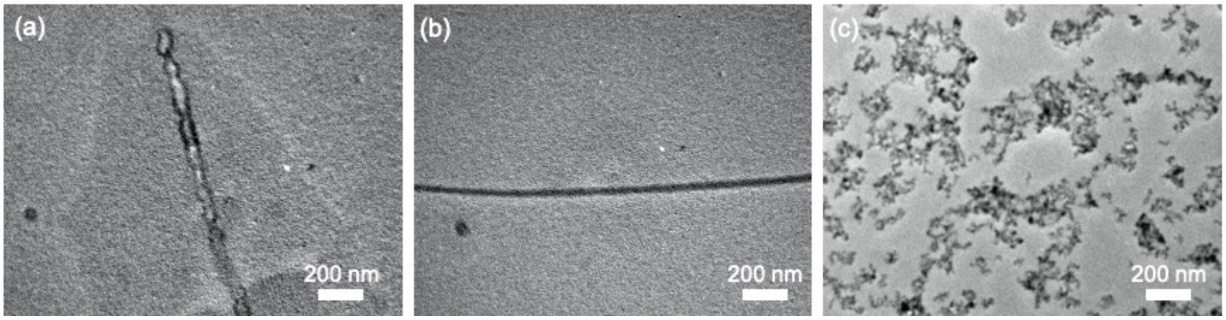

Figure S6. TEM images of isolated ZIF-8 10 nm nano-structures from reaction conditions (a)-(c) in Table S6.

Table S7. Synthesis condition optimization for ZIF-8 nano-structures using PCTE templates with 200 nm pore sizes. 2-MIM: 2-Methylimidazole. $Zn(NO_3)_2$ is the Zn^{2+} source.

Attempts	*[Zn^{2+}] Conc.(M)*	*[2-MIM] Conc. (M)*	*Reaction Time (h)*
(a)	0.025	0.5	4
(b)	0.025	1	4
(c)	0.025	2	4

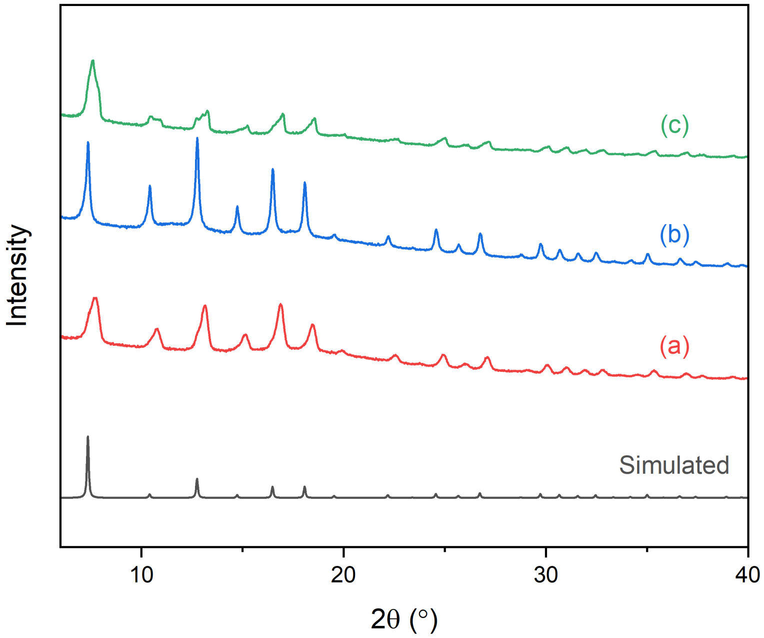

Figure S7. PXRD profiles of post-synthesis templates using conditions listed in Table S7.

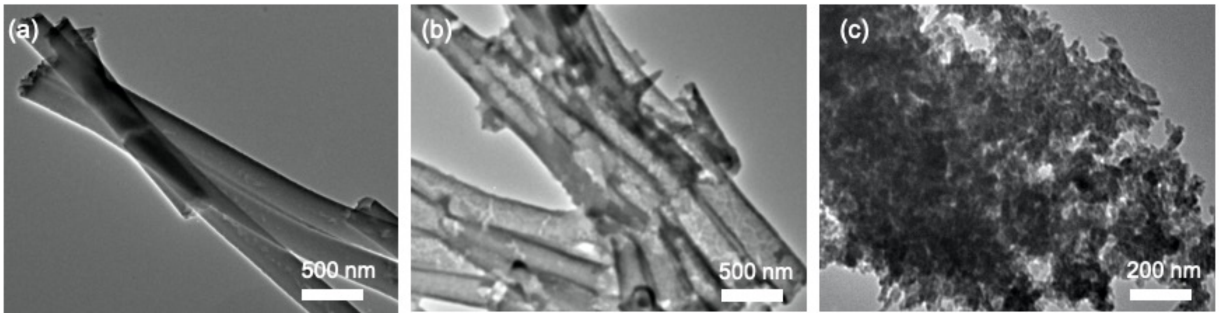

Figure S8. TEM images of isolated ZIF-8 200 nm nano-structures from reaction conditions (a)-(c) in Table S7.

Table S8. Synthesis condition optimization for ZIF-8 micro-structures using PCTE templates with 2 µm pore sizes. 2-MIM: 2-Methylimidazole. $Zn(NO_3)_2$ is the Zn^{2+} source.

Attempts	*[Zn^{2+}] Conc.(M)*	*[2-MIM] Conc. (M)*	*Reaction Time (h)*
(a)	0.025	1	4
(b)	0.06	1	4
(c)	0.1	1	4
(d)	0.5	1	4
(e)	1	1	4
(f)	1.5	1	4
(g)	2	1	4
(h)	0.025	2	4
(i)	0.06	2	4
(j)	0.1	2	4
(k)	0.5	2	4

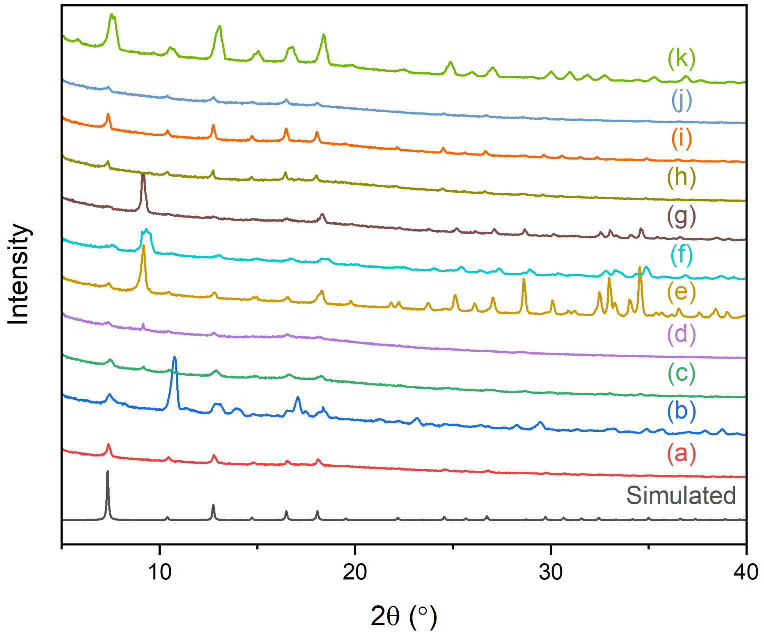

Figure S9. PXRD profiles of post-synthesis templates using conditions listed in Table S8.

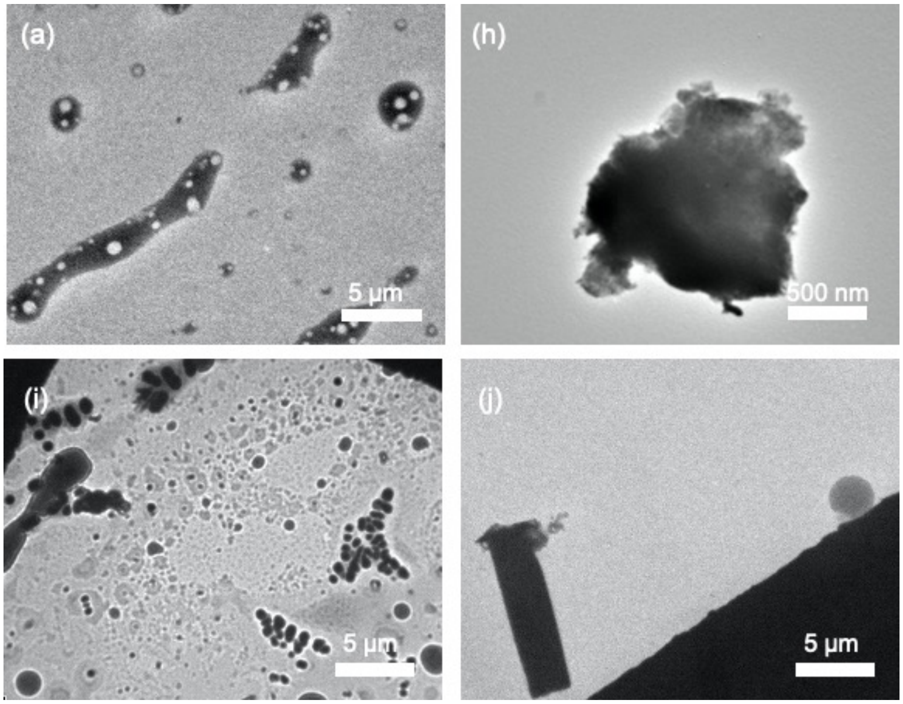

Figure S10. TEM images of isolated ZIF-8 2 μm micro-structures from reaction conditions (a), (h), (i), (j) in Table S8.

Table S9. Synthesis condition optimization for ZIF-8 micro-structures using PCTE templates with 10 μm pore sizes. 2-MIM: 2-Methylimidazole. $Zn(NO_3)_2$ is the Zn^{2+} source.

Attempts	*[Zn^{2+}] Conc.(M)*	*[2-MIM] Conc. (M)*	*Reaction Time (h)*
(a)	0.025	1	4
(b)	0.06	1	4
(c)	0.1	1	4
(d)	0.5	1	4
(e)	1	1	4
(f)	0.025	2	4
(g)	0.06	2	4
(h)	0.1	2	4
(i)	0.5	2	4
(j)	1	2	4

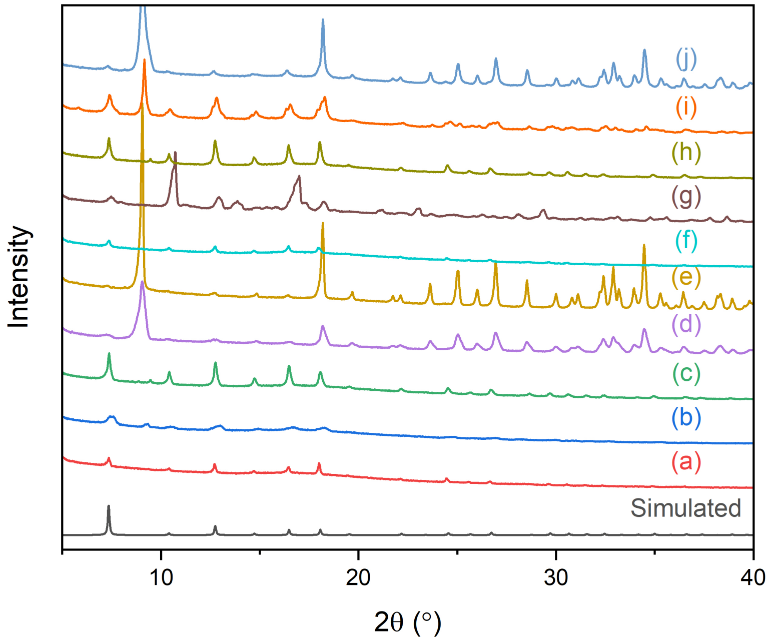

Figure S11. PXRD profiles of post-synthesis templates using conditions listed in Table S9.

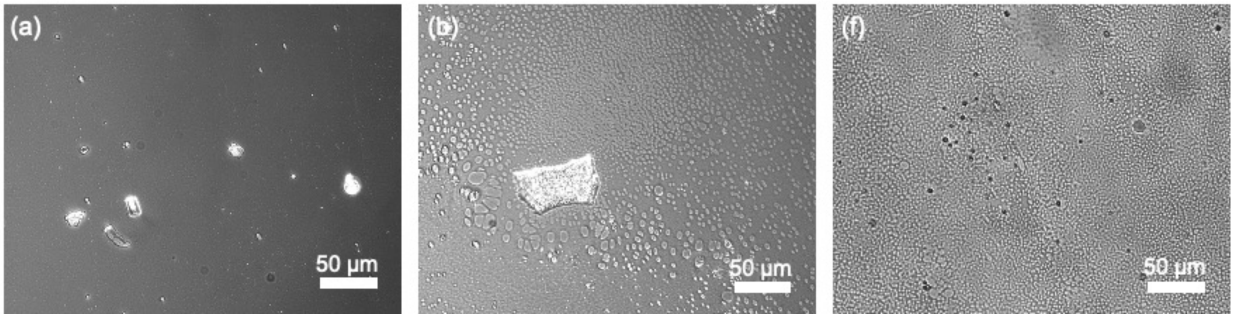

Figure S12. Optical microscopy images of isolated ZIF-8 10 μm micro-structures from reaction conditions (a), (b), (f) in Table S9.

Table S10. Synthesis condition optimization for ZIF-8 micro-structures using PCTE templates with 20 μm pore sizes. 2-MIM: 2-Methylimidazole. $Zn(NO_3)_2$ is the Zn^{2+} source.

Attempts	*[Zn^{2+}] Conc.(M)*	*[2-MIM] Conc. (M)*	*Reaction Time (h)*
(a)	0.025	1	4
(b)	0.025	2	4

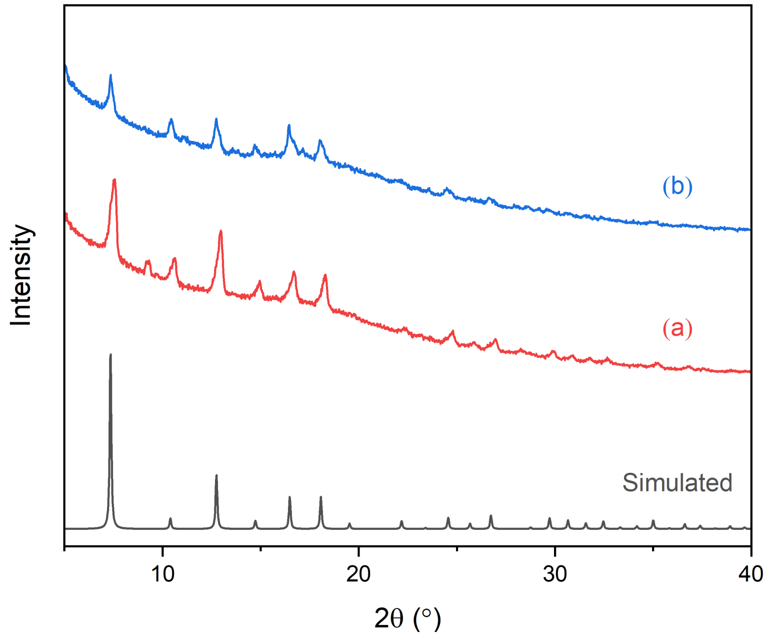

Figure S13. PXRD profiles of post-synthesis templates using conditions listed in Table S10.

Table S11. Synthesis condition optimization for ZIF-67 nano-structures using PCTE templates with 10 nm pore sizes. 2-MIM: 2-Methylimidazole. $Co(NO_3)_2$ is the Co^{2+} source.

Attempts	*[Co^{2+}] Conc.(M)*	*[2-MIM] Conc. (M)*	*Reaction Time (h)*
(a)	0.025	1	4
(b)	0.042	1	4
(c)	0.06	1	4
(d)	0.025	2	4
(e)	0.042	2	4
(f)	0.06	2	4

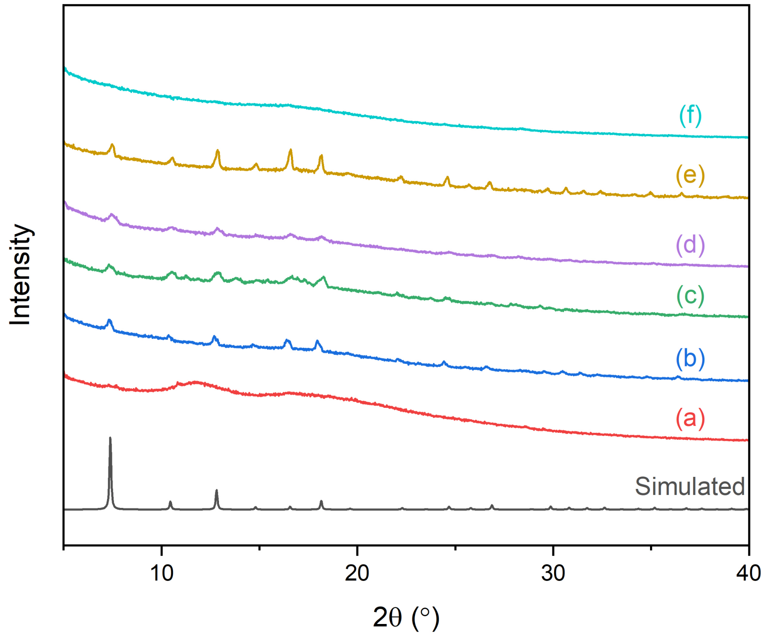

Figure S14. PXRD profiles of post-synthesis templates using conditions listed in Table S11.

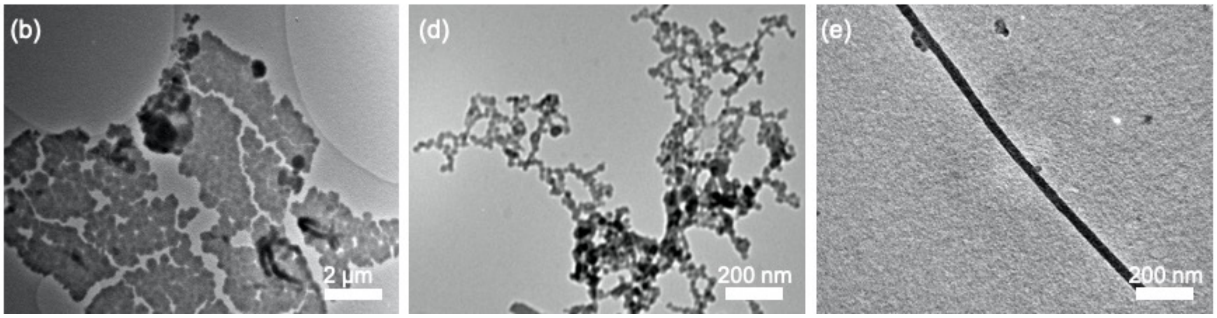

Figure S15. TEM images of isolated ZIF-67 10 nm nano-structures from reaction conditions (b), (d), (e) in Table S11.

Table S12. Synthesis condition optimization for ZIF-67 nano-structures using PCTE templates with 30 nm pore sizes. 2-MIM: 2-Methylimidazole. $Co(NO_3)_2$ is the Co^{2+} source.

Attempts	*[Co^{2+}] Conc.(M)*	*[2-MIM] Conc. (M)*	*Reaction Time (h)*
(a)	0.008	1	1
(b)	0.025	1	1
(c)	0.06	1	1
(d)	0.1	1	1
(e)	0.025	0.5	1
(f)	0.025	0.75	1
(g)	0.025	2	1

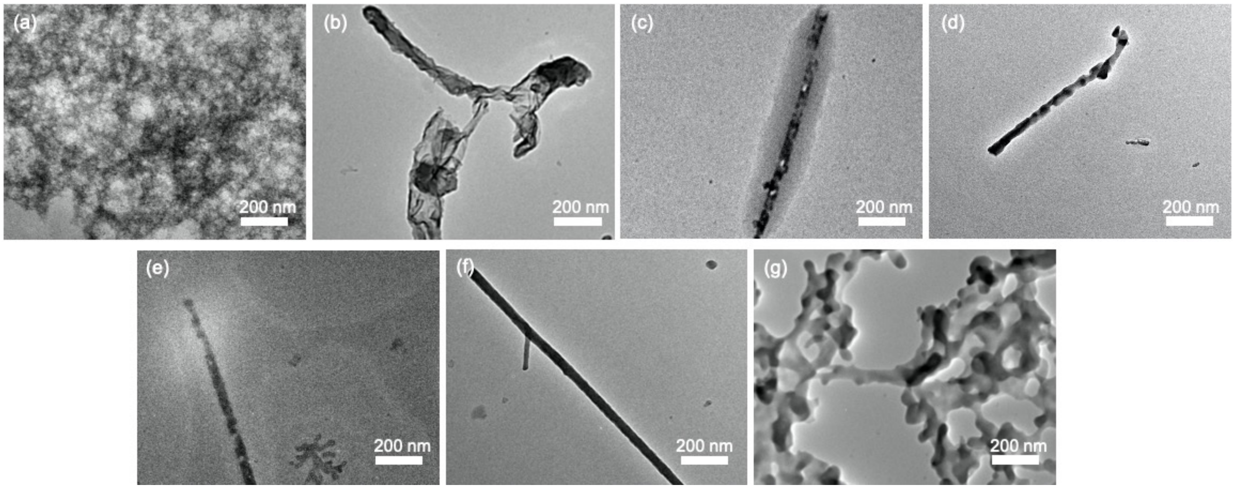

Figure S16. TEM images of isolated ZIF-67 30 nm nano-structures from reaction conditions (a)-(g) in Table S12.

Table S13. Synthesis condition optimization for ZIF-67 nano-structures using PCTE templates with 100 nm pore sizes. 2-MIM: 2-Methylimidazole. $Co(NO_3)_2$ is the Co^{2+} source.

Attempts	*[Co^{2+}] Conc.(M)*	*[2-MIM] Conc. (M)*	*Reaction Time (h)*
(a)	0.025	2	1
(b)	0.042	2	1
(c)	0.06	2	1

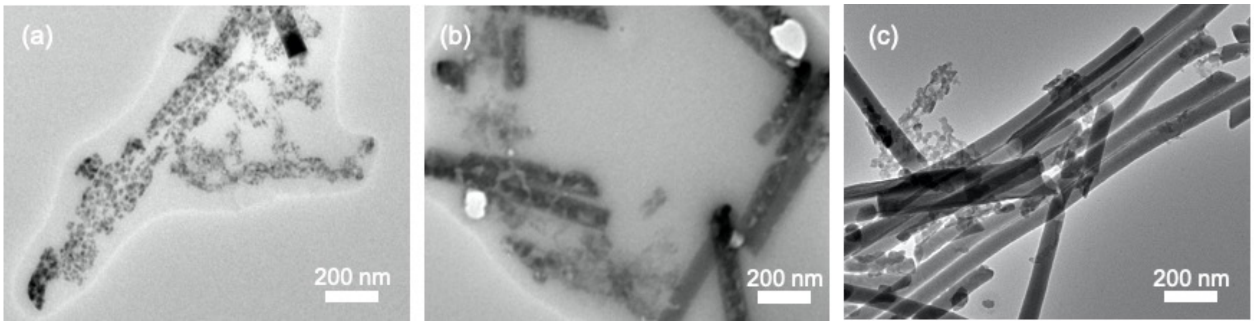

Figure S17. TEM images of isolated ZIF-67 100 nm nano-structures from reaction conditions (a)-(c) in Table S13.

Table S14. Synthesis condition optimization for ZIF-67 nano-structures using PCTE templates with 200 nm pore sizes. 2-MIM: 2-Methylimidazole. $Co(NO_3)_2$ is the Co^{2+} source.

Attempts	*[Co^{2+}] Conc.(M)*	*[2-MIM] Conc. (M)*	*Reaction Time (h)*
(a)	0.025	0.5	1
(b)	0.06	2	1
(c)	0.06	2	4

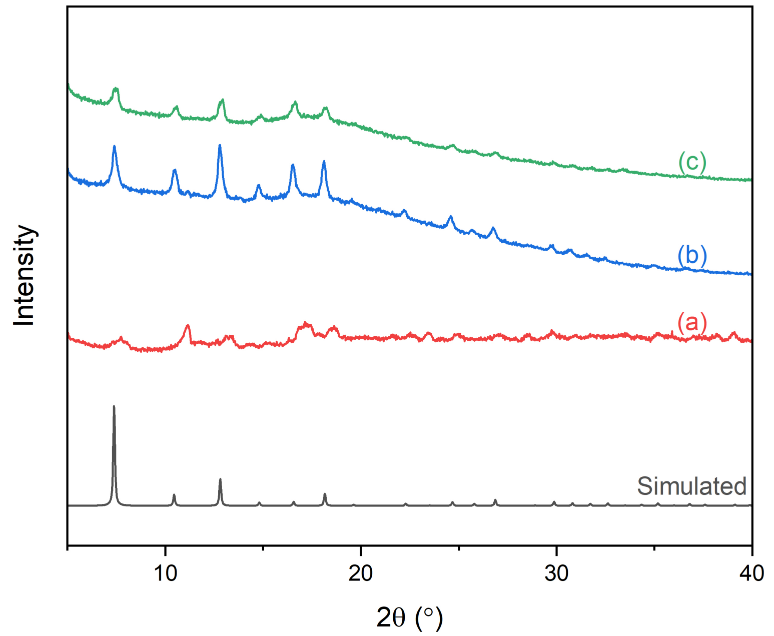

Figure S18. PXRD profiles of post-synthesis templates using conditions listed in Table S14.

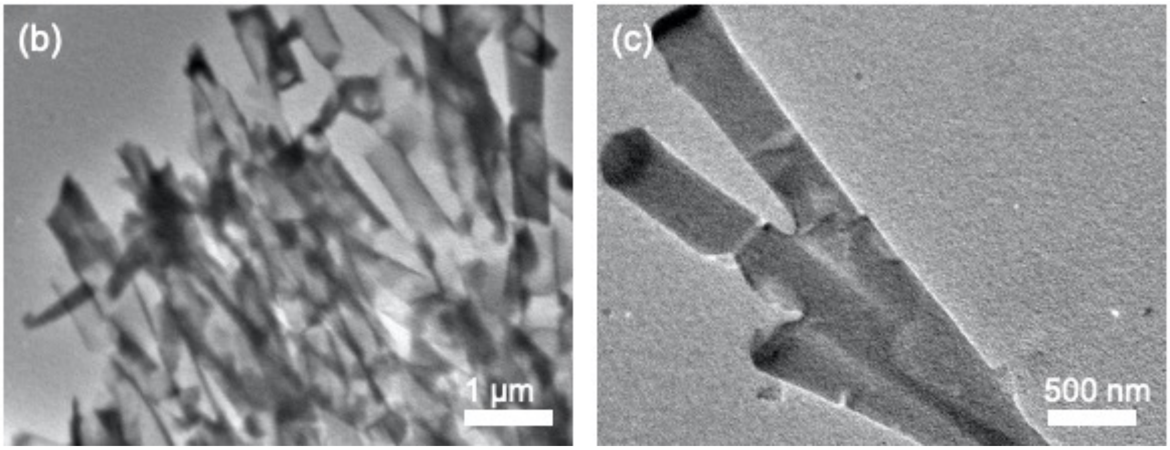

Figure S19. TEM images of isolated ZIF-67 200 nm nano-structures from reaction conditions (b)-(c) in Table S14.

Table S15. Synthesis condition optimization for ZIF-67 micro-structures using PCTE templates with 10 µm pore sizes. 2-MIM: 2-Methylimidazole. $Co(NO_3)_2$ is the Co^{2+} source.

Attempts	*[Co^{2+}] Conc.(M)*	*[2-MIM] Conc. (M)*	*Reaction Time (h)*
(a)	0.025	1	4
(b)	0.025	1	6
(c)	0.06	1	4
(d)	0.06	1	6
(e)	0.06	2	6

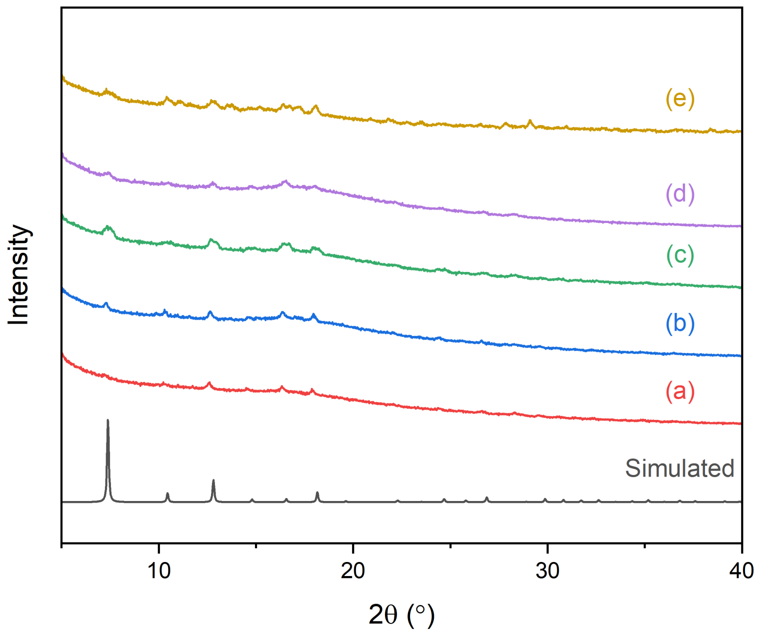

Figure S20. PXRD profiles of post-synthesis templates using conditions listed in Table S15.

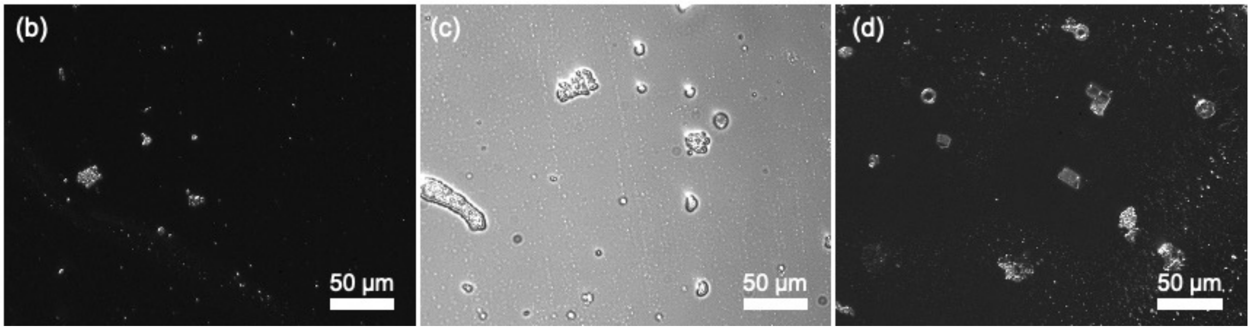

Figure S21. Optical microscopy images of isolated ZIF-67 10 μm micro-structures from reaction conditions (b)-(d) in Table S15.

Table S16. Synthesis condition optimization for ZIF-67 micro-structures using PCTE templates with 20 μm pore sizes. 2-MIM: 2-Methylimidazole. $Co(NO_3)_2$ is the Co^{2+} source.

Attempts	*[Co^{2+}] Conc.(M)*	*[2-MIM] Conc. (M)*	*Reaction Time (h)*
(a)	0.025	2	4
(b)	0.025	2	6
(c)	0.06	2	4
(d)	0.06	2	6
(e)	0.1	2	4
(f)	0.1	2	6

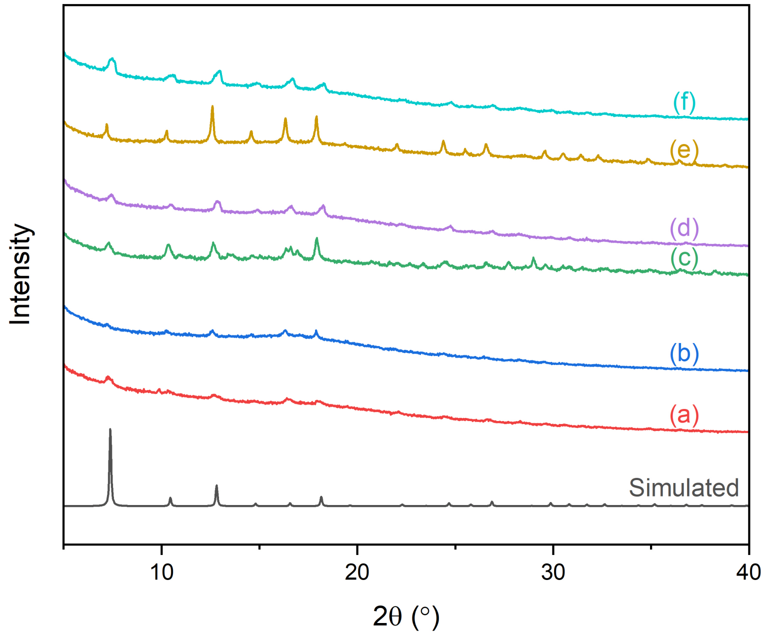

Figure S22. PXRD profiles of post-synthesis templates using conditions listed in Table S16.

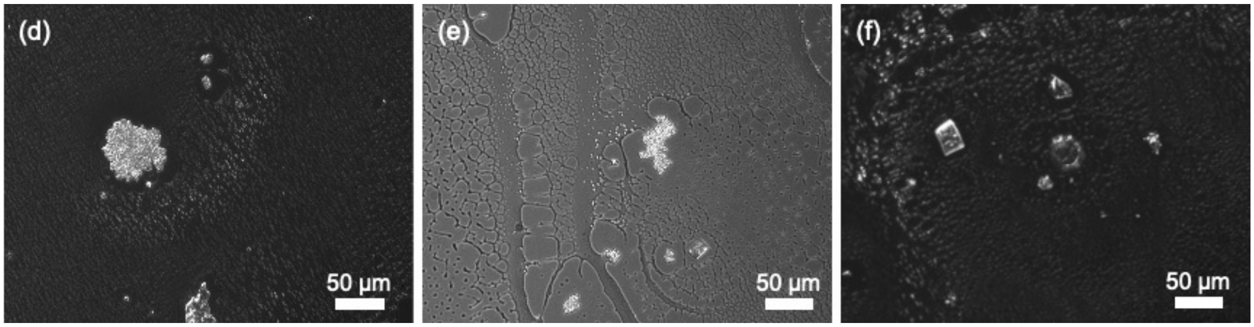

Figure S23. Optical microscopy images of isolated ZIF-67 20 μm micro-structures from reaction conditions (d), (e), (f) in Table S16.

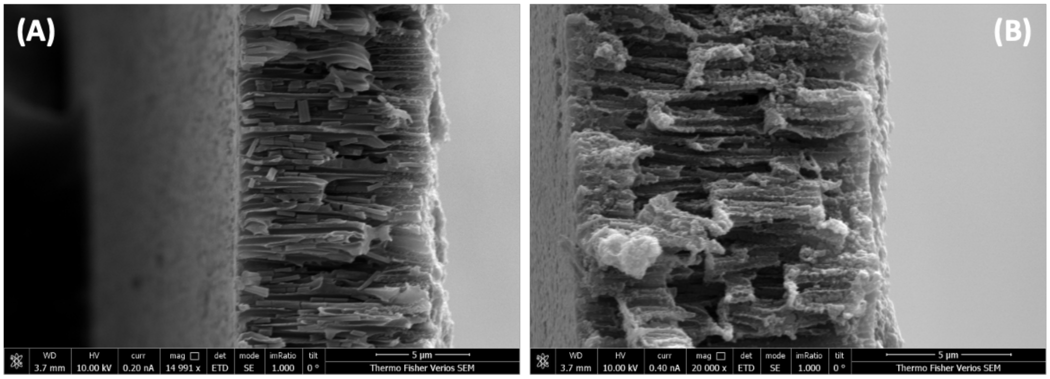

Figure S24. Cross-section SEM images of ZIF-8 post-synthesis templates of (A) PCTE and (B) PETE templates of 200 nm pore sizes. Reactions conditions are that optimized for ZIF-8 using 200 nm PCTE templates. Left side facing 1-octanol during synthesis in both images.
